# Effect of Kefir on Soybean Isoflavone Aglycone Content in Soymilk Kefir

**DOI:** 10.3389/fnut.2020.587665

**Published:** 2020-12-16

**Authors:** Minke Yang, Xiaojuan Yang, Xiaoqu Chen, Jie Wang, Zhenlin Liao, Li Wang, Qingping Zhong, Xiang Fang

**Affiliations:** College of Food Science, South China Agricultural University, Guangzhou, China

**Keywords:** kefir, whole genome sequencing, soy bean cultivar, isoflavone aglycones, biotransformation, soymilk

## Abstract

Kefir is a traditional fermented milk originating in the Caucasus area and parts of Eastern Europe. In this study, the kefir culture, which is modified upon the addition of lactic acid bacteria (LAB) cells, specifically for soymilk kefir fermentation with the highest capacity of isoflavone biotransformation, was successfully produced, and the metagenomics composition of soymilk or milk fermented using these kefir cultures was investigated. The metagenome analysis showed that the microbiota of kefir in M-K (milk inoculated with kefir), SM-K (equal volumes of soymilk and milk inoculated with kefir), and S-K (pure milk inoculated with kefir) were related to the addition of soymilk or not. Furthermore, the HPLC chromatogram revealed that Guixia 2 (Guangzhou, China) may be a good source of soymilk kefir fermentation due to its high isoflavone aglycone content (90.23 ± 1.26 μg/g in daidzein, 68.20 ± 0.74 μg/g in genistein). Importantly, the starter culture created by adding 1.5 g probiotics (Biostime®, Guangzhou, China) to Chinese kefir showed a significant increase in the levels of isoflavone aglycones (72.07 ± 0.53 μg/g in isoflavone aglycones). These results provided insight into understanding the suitable soybean cultivar and starter cultures, which exhibit promising results of isoflavone biotransformation and flavor promotion during soymilk kefir fermentation.

## Introduction

Kefir, a fermented dairy beverage, is characterized by an acidic, mildly alcoholic flavor and creamy consistency (1). It originated in the Caucasian region and became popular in Tibet and Mongolia (2). Traditionally, kefir grains play a natural starter culture role in the production of kefir (3). They are gelatinous, white-to-light-yellow clusters, with a small, irregular cauliflower-like shape (4). As a mixed microflora, these grains are composed of an inert polysaccharide/protein matrix containing different lactic acid bacteria (LAB), yeasts, and occasionally acetic acid bacteria (AAB) in a complex symbiotic association (5), which are responsible for lactic-alcoholic fermentation (6). In general, *Kluyveromyces, Saccharomyces, Lactobacillus, Lactococcus, Leuconostoc*, and *Acetobacter* are predominant species in starter grains (7). Historically, kefir has been considered a beneficial food, with probiotic microorganisms and functional organic substances. The consumption of this fermented beverage has been recognized for a variety of health properties, such as antibacterial, antifungal, anti-allergic, and anti-inflammatory properties (8). Furthermore, some of the bioactive compounds in the kefir, including polysaccharides (kefiran), peptides, amino acids, ethanol, CO_2_, acetaldehyde, acetoin, diacetyl, folic acid, calcium, and vitamins (B_1_, B_12_, and K), may contribute to these health-promoting and antimicrobial effects (9–12).

Soybean and its derivatives, which have great potential for applications in the functional food industry, are considered rich in proteins, isoflavones, and oligosaccharides (13). However, the nutritional and phytochemical compounds of soybean may vary considerably depending on cultivar, which was significantly positively correlated with the quality of soymilk. For example, some cultivars show high contents of protein but are low in ash and total solids in seeds, which meet the quality requirements of soymilk processing. Others with removal of lipoxygenase-2 show flavor improvement (14, 15). Isoflavones, in particular, have recently received more attention due to their antioxidant and estrogenic effects. Isoflavones occurred in four different chemical structures: aglycones or the free forms (daidzein, genistein, and glycitein); 7-O-β-D-glucosides or β-glucosides (daidzin, genistin, and glycitin); 6″-O-acetyl-7-O-β-D-glucosides or acetyl glucosides (acetyl daidzin, acetyl genistin and acetyl glycitin); and 6″-O-malonyl-7-O-β-D-glucosides or malonyl glucosides (malonyl daidzin, malonyl genistin, and malonyl glycitin) (16). With the lower molecular weight, aglycone forms showed improved diffusion and absorption in the human gut, resulting in better absorption than conjugates (17). Importantly, the isoflavone content may vary because of narrow genetic diversity in wild soybean (18, 19). It is also reported that lower isoflavone concentrations are generally presented in early rather than late maturing soybean cultivars (20). In addition to the genetic factor, environmental factors also influence the isoflavone content and other components (21, 22). For instance, the malonylated isoflavone glycosides were thermally unstable and were easily converted into their corresponding isoflavone glycosides under high temperature (23).

Many studies have indicated that soymilk fermented with kefir may be beneficial to human health (24–26). After fermentation, the content of aglycone isoflavone and total phenolic in soymilk kefir multiplied *in vitro* digestive system simulation (16). The populations of probiotics in the intestinal ecosystem tended to be improved during the period of fermented soymilk intake (27). The soymilk fermented with kefir and a *Bifidobacterium longum* strain had a high rate of the desired volatile aroma compounds (acetoin, diacetyl) and a low rate of the undesired compounds (1-octen-3-ol, 2-penten-1-ol, (E)-2-heptenal, 1-hexanol) (28). However, the kefir studies showed a high number of LAB strains but a low number of species (29). Importantly, some species that are not represented or exist only in very low numbers exhibit strong probiotic activity (30, 31). This phenomenon constrains the healthy function of kefir and their byproducts. Most isoflavones exist as glycosylated forms and, to a lesser extent, as aglycones (32). β-Glucosidase, which is naturally present in soybean and considered to be the key enzyme during the hydrolysis of β-glucosides into aglycones, is produced by various microorganisms from kefir (17, 26).

The selection of suitable soybean cultivar and starter for soymilk kefir has been little investigated. In the current work, the effect of soybean cultivars on soymilk fermentation was determined by using the kefir preserved in our laboratory (Kefir C), followed by metagenomics analysis. Moreover, new kefir starters were produced by the addition of lactic acid bacteria (LAB) to kefir from three different sources, including Kefir C, Chinese kefir (Kefir A), and Caucasus kefir (Kefir B). Then, high-performance liquid chromatography (HPLC) was used to examine their effectiveness in increasing the biotransformation of isoflavone to determine the most suitable starter for soymilk kefir fermentation.

## Materials and Methods

### Soybean Materials

Ten different soybean cultivars, which were grown in Guangdong and Hunan, were selected for this research. The sample information is shown in Table 1.

**Table 1:** Description of the soybean materials.

**Code**	**Cultivar name**	**Origin**
1	Huaxia 6	Xintian, Hunan
2	Huaxia 3	Yingde, Guangdong
3	Huaxia 9	Yingde, Guangdong
4	Guixiadou 2	Yingde, Guangdong
5	Huaxia 10	Guangzhou, Guangdong
6	Huachun 2	Guangzhou, Guangdong
7	Huachun 5	Guangzhou, Guangdong
8	Huaxia 9	Guangzhou, Guangdong
9	Guixiadou 2	Guangzhou, Guangdong
10	Wayao	Guangzhou, Guangdong

### Preparation of Kefir Starters

The three water kefirs used in this study are from different sources. Chinese kefir (Kefir A) and Caucasus kefir (Kefir B) were purchased from the Taobao website, and our laboratory provided the third (Kefir C). These kefirs were inoculated at the rate of 3.3% (V/V) in sterilized whole milk, which was composed of whole milk powder (Fonterra™, Auckland, New Zealand) with water (10 wt %/volume), and then incubated at 25°C for 24 h. After incubation, the product was filtrated through a sieve to remove the clotted milk and the grains were washed gently with sterile water. This activation step was repeated three times.

After the activation, three types of modified kefir were manufactured using activated kefir grains. Specifically, 5 mL of fermented milk (Bright Dairy & Food Co., Ltd., Shanghai, China), which contained mixed LAB strains of *Bifidobacterium lactis, Lactobacillus plantarum* ST-111, *L. bulgaricus*, and *Streptococcus thermophiles*, were added into activated kefir grains during the 10-days subculture (named Starter 1, sample S1-A, S1-B, and S1-C). Following incubation, 1/2 kefir grains were filtered through a sieve to remove the coagulated milk and then rinsed with sterile water. The remaining kefir liquid product (kefir sample 1) was freeze-dried on the 12th (sample A11, B11, C11) and 27th (sample A12, B12, C12) days of subculture, using the Freeze Drying Machine (CHRIST ALPHA, Guangxi, China). In another group, the isolated kefir grains were activated as described above with the addition of 1.5 g probiotics (Biostime®, Guangzhou, China), which contains *L. helveticus, B. bifidum, B. infantis* (named Starter 2, samples S2-A, S2-B, and S2-C), during the 10-days incubation. On day 12 (samples A21, B21, C21) and 27 (samples A22, B22, C22), the kefir filtrate was lyophilized into powder. The stock solution without addition as the control group and the sterilized whole milk were renewed daily.

### Fermentation of Soymilk Kefir

The dry soybeans (1 kg) were blanched in hot water for 3 min and then soaked in 0.1% NaHCO_3_ at room temperature for 12 h, with a bean-to-water ratio of 1:9 (w/w). The soybeans were then drained and ground with 6.5 times (w/w) distilled water using a high-speed blender (SUPOR, Zhejiang, China). The resulting slurry was filtered through a sieve to separate the soymilk from the residue.

Soymilk was sterilized in an ultra-high temperature sterilization de-fishing machine (RUIPAI, Shanghai, China) at sterilizing temperature (135–140°C), vacuum degree (0.01–0.025 mpa), and then concentrated in a three-effect evaporator (LiJie, Zhejiang, China) at the exit concentration (43–46%) and exit temperature (45°C). The drying process was performed in a lab-scale spray dryer (YaChen, Shanghai, China) under the following conditions: inlet-air temperature (170°C), outlet air temperature (93–94°C), and air pressure (10 mpa). The spray-dried soymilk powders were collected and packed in airtight bags and stored in the refrigerator until further analysis.

Kefirs C, S1, and S2 were filtered through a sieve aseptically, then inoculated at the rate of 3.3% (V/V) in sterilized whole milk (10 wt %/volume), and then incubated at 25°C for 24 h. Pure water at a temperature of 95°C was added to the soya bean flour (soya bean flour/water = 1:6.5; m/v), milk powder (milk powder/water = 1:10; m/v), and sugar (sugar/water = 1:10; m/v). Next, milk, pure soymilk, and a mixture of the two (1:1; V/V) were inoculated with Kefir C culture (1%, m/v, named samples M-K, SM-K, and S-K) for genomic sequencing after incubating at 25°C for 24 h. A starter consisting of a mixture of equal volumes of soymilk and milk was inoculated with 5% (m/v) kefir S1 and S2 culture and incubated at 25°C for 24 h. After fermentation, samples were stored at 4°C for 24 h. Fermentation was not performed in the soymilk control. The parameters mentioned above, such as inoculate percentage and fermentation temperature (unpublished work), were optimized in our previous studies, which reported that under these conditions there was a maximum conversion of β-glucoside to aglycone isoflavones, taking into account the low temperature and high altitude of the kefir birthplace, the Caucasus (17, 33).

### Metagenomics Analysis

The metagenomics DNA was extracted and purified using a High Pure PCR Template Preparation Kit (Roche, Basel, Switzerland), according to the manufacturer's instructions. Specifically, 300 μL of M-K, SM-K, and S-K were centrifuged and treated with Iysozyme, chemical buffer, and proteinase K, followed by phenol/chloroform/isoproponal extraction as described previously (34). Sodium acetate/glacial acetic acid/ethanol was used to purify the 300 μL DNA eluates. The metagenomics DNA were obtained and sent to the Institute of Microbiology Epidemiology (Beijing, China) for sequencing.

### Sensory Analysis

To determine the suitable starter for soymilk kefir, sensory evaluation was carried out by seven trained panelists who were familiar with soymilk fermentation sensory methodology. They were asked to score for color, smell, taste, and texture of the samples, which was based on the method reported previously (35). Before assessing the samples, ~10 g of each soymilk kefir was loaded into plastic cups, tempered to 20°C, and coded with a random three-digit number. The evaluation was carried out under proper lighting. Panelists were given water to rinse their mouths between tests. All samples were scored from 0 (lowest) to 10 (highest) for sensory attributes.

### Determination of the Contents of the Different Isoflavones in Soymilk Kefir

After soymilk kefir fermentation, samples were frozen at −40°C and subsequently lyophilized to powder. The extraction of isoflavones was prepared by weighing 0.5 g of sample and mixing with 5 mL of 80% HPLC-grade methanol in a 15-mL centrifuge tube. The mixture was allowed to stand in an ultrasonic bath for 3 h at 50°C (Kunshan Ultrasonic Instrument, Jiangsu, China), centrifuged at 12,000 *g* for 10 min at 4°C (USTC ZONKIA, Anhui, China). The resulting supernatant was passed through a 0.22-μm syringe filter (Jinteng, Tianjin, China) for HPLC analysis. Soymilk with the same preparation was used as a control. Each experiment was characterized by the mean in triplicate.

The isoflavones were separated and quantified in an HPLC system (Shimadzu, Tokyo, Japan), equipped with wavelength detector (PDA-100), and coated C18 column (250 nm × 4.6 nm, 5 μm). Then 10 μL isoflavones were eluted with a mobile phase consisting of 0.1% (v/v) formic acid in acetonitrile (solvent A) and 0.1% (v/v) formic acid in water (Solvent B), and at a flow rate of 1.000 mL/min at 34°C. Eluted isoflavones were detected by their absorbance at 260 nm. For the construction of calibration curves, the following standard isoflavones were used: acetyl- and malonyl-conjugated glucosides (Wako Pure Chemical Industries, Ltd., Osaka, Japan), β-glucosides, and aglycones (Sigma-Aldrich Co., St. Louis, MO, USA).

### Statistical Analysis

Experimental results were recorded by means ± standard deviation (SD) of triplicate determinations. Statistical analysis was subjected to one-way ANOVA and *T*-test using SPSS 26.0 (SPSS Inc., Chicago, IL, USA) and Graphpad Prism 8.4.3 (Graphpad, San Diego, CA, USA).

## Results

### Metagenomics Analysis of the Microbial Composition From M-K, SM-K, and S-K

**Figure 1:**
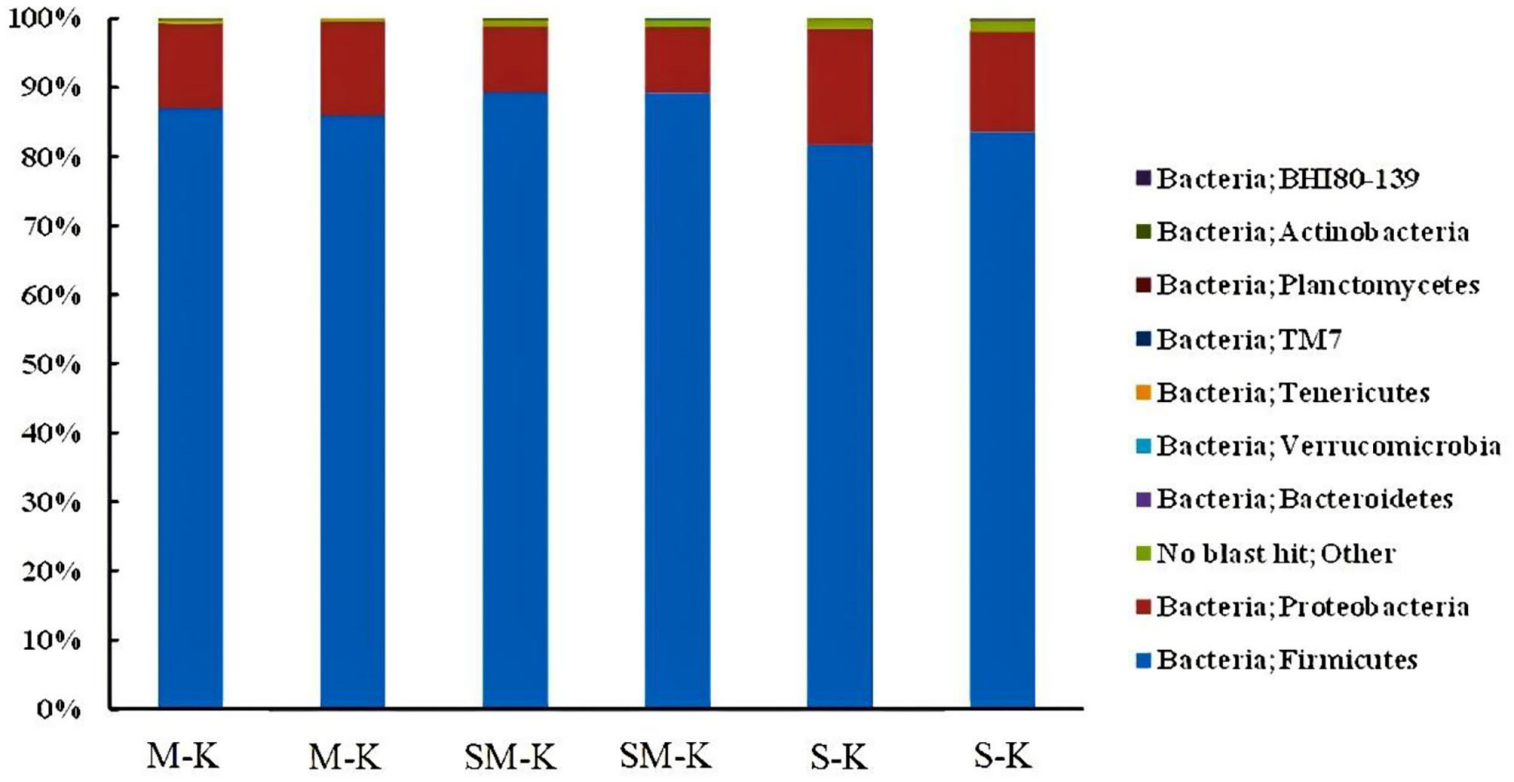
Relative abundance (%) of metagenomes assigned to different phyla detected in M-K, SM-K, and S-K. M-K: Milk was inoculated with kefir; SM-K: equal volumes of soymilk and milk were inoculated with kefir; S-K: pure milk was inoculated with kefir.

The phylogenetic classification of bacterial sequences is summarized in Figure 1. The sequences were distributed among three bacterial phyla, Firmicutes, Proteobacteria, and Bacteroidetes. Notably, Firmicutes dominated in all samples, corresponding to 86.42 ± 0.007%, 89.21 ± 0.007%, 82.58 ± 0.013% in M-K, SM-K, and S-K, respectively. The second dominated bacterial phylum was Proteobacteria, representing an average of 13.00 ± 0.009%, 9.64 ± 0.0009%, and 15.80 ± 0.016% of the population in M-K, SM-K, and S-K, respectively. The richness of Firmicutes and Proteobacteria was significantly different from three kinds of fermented beverages (^*^p < 0.05). However, the difference in Bacteroidetes and others were not statistically significant (*p* > 0.05) among groups. It is worth noting that higher diversity in the bacterial phyla presented when soymilk was added in the fermentation matrix.

**Figure 2:**
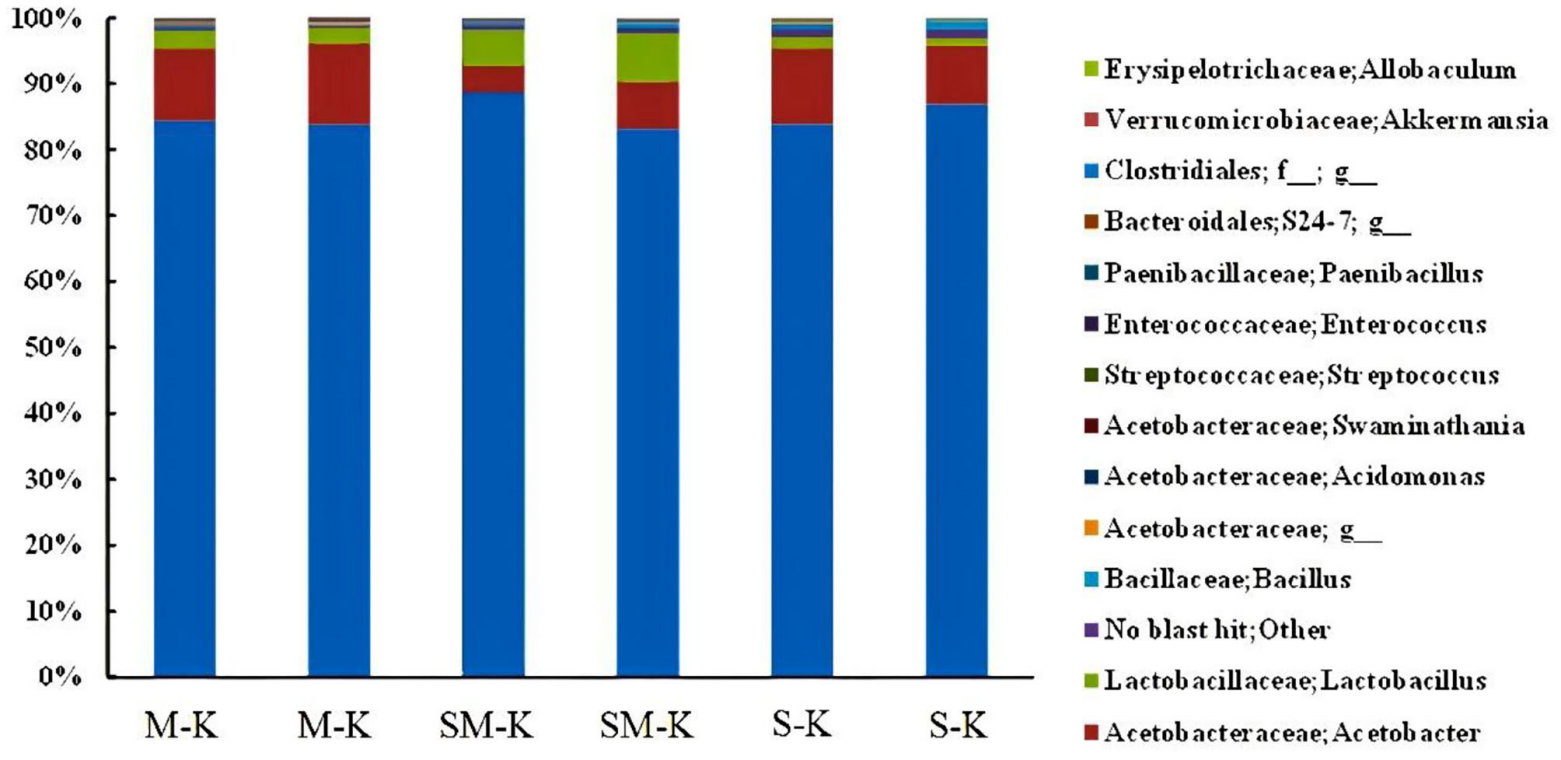
Relative abundance (%) of metagenomes assigned to different genera detected in M-K, SM-K, and S-K. M-K: Milk was inoculated with kefir; SM-K: equal volumes of soymilk and milk were inoculated with kefir; S-K: pure milk was inoculated with kefir.

Moreover, our results showed that there were major and minor genera in every fermented beverage sample (Figure 2). Specifically, *Acetobacter* dominated (more than 85%), followed by *Lactobacillus* and *Streptoccus*. The richness of *Lactobacillus* varied dramatically in three fermented samples (2.50 ± 0.002% in M-K, 6.34 ± 0.015% in SM-K, 1.41 ± 0.005% in S-K). Similarly, higher diversity in the bacterial species presented when soymilk was added in the fermentation matrix.

### Sensory Analysis of Soymilk Kefir

In the sensory evaluation, the five starters with the highest scores (sample Kefir B, S1-A, S2-B, S2-A, S1-B, and S1-C) were used as mixed starters for further soymilk kefir fermentation (Table 2). Interestingly, the soymilk kefir fermented using other starters always generated more bubbles and heavier acidity, which affected the appearance and taste of the product.

**Table 2:** Sensory analysis of the soymilk kefir fermented with different kefir starters.

**Sample**	**B**	**S1-A**	**S2-B**	**S2-A**	**S1-B**	**S1-C**	**A**	**C**	**S2-C**
Score	54.30 ± 2.97^a^	53.57 ± 3.27^a^	52.87 ± 3.04^a^	52.27 ± 3.04^a^	52.03 ± 0.25^a^	51.77 ± 0.40^a^	50.33 ± 2.04^a^	50.50 ± 1.30^a^	49.43 ± 2.40^b^

Mean ±standard deviation; n = 3. Different superscript letters indicate significant differences among cultivars (*p < 0.05). A: soymilk fermented with kefir A; B: soymilk fermented with kefir B; C: soymilk fermented with kefir C; S1-A: soymilk fermented with the addition of 5 mL fermented milk to kefir A; S2-A: soymilk fermented with the addition of 1.5 g probiotics to kefir A; S1-B: soymilk fermented with the addition of 5 mL fermented milk to kefir B; S2-B: soymilk fermented with the addition of 1.5 g probiotics to kefir B; S1-C: soymilk fermented with the addition of 5 mL fermented milk to kefir C. Different superscript letters (a and b) indicate significant differences in sensory score among different soymilk kefir (*p < 0.05).

### Changes in Isoflavone Contents in Soymilk Kefir With 10 Kinds of Soybean Cultivars

**Figure 3:**
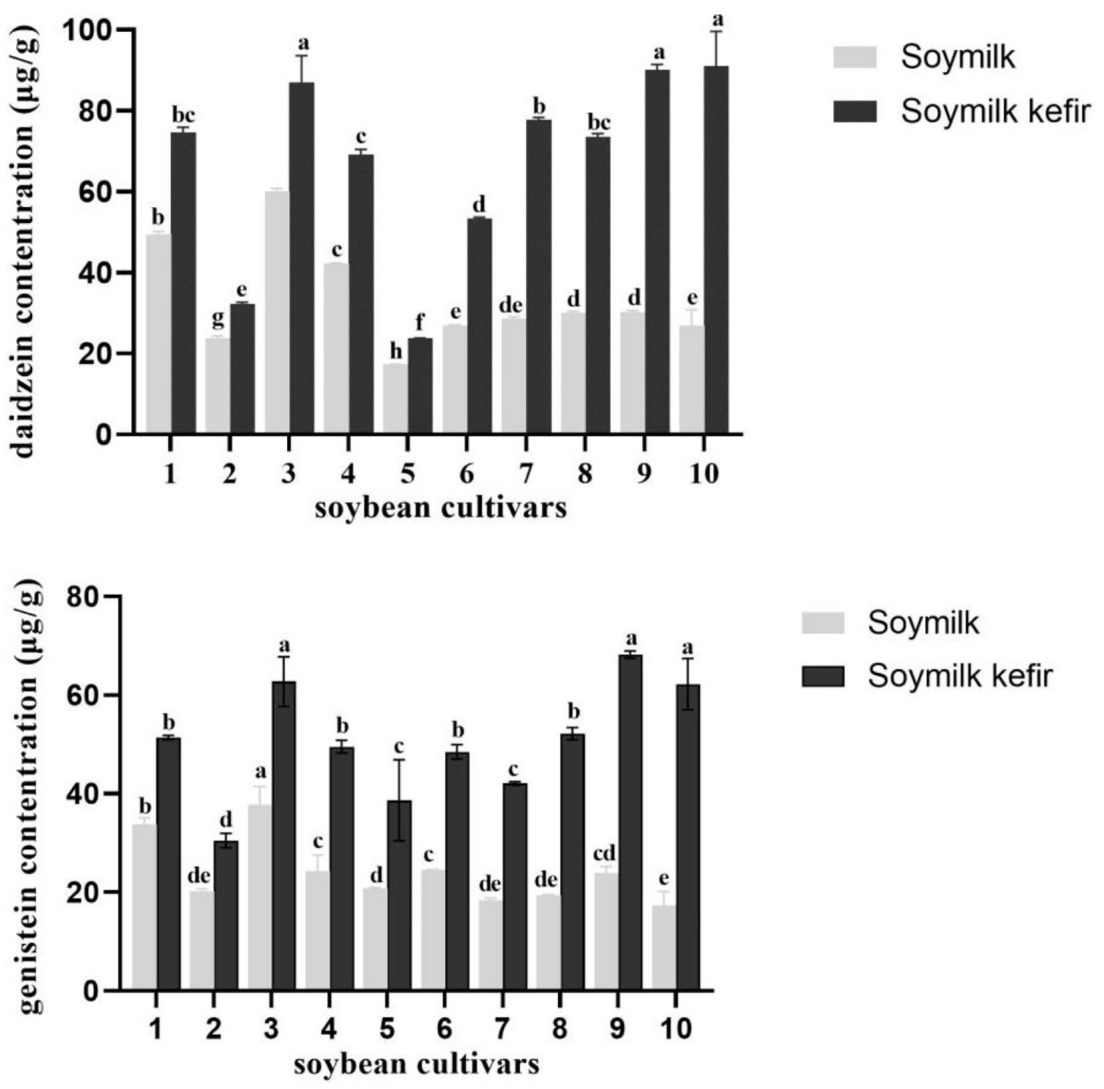
The concentration of daidzein and genistein in soymilk kefir made from 10 soybean cultivars and kefir C. Different superscript letters indicate significant differences among cultivars (*p < 0.05). (1) Huaxia 6 Soybean, (2) Huaxia 3 Soybean, (3) Huaxia 9 Soybean, (4) Guixiadou 2 Soybean (Yingde, Guangdong), (5) Huaxia 10 Soybean, (6) Huachun 2 Soybean, (7) Huachun 5 Soybean, Huaxia 9 Soybean, (9) Guixiadou 2 Soybean (Guangzhou, Guangdong), (10) Wayao Soybean.

Table 3 shows the concentration of isoflavone aglycones in 10 soybean varieties. Importantly, we found dramatic variations in the isoflavone content in the 10 soybean cultivars. The levels of aglycone isoflavone daidzein content were 17.35–60.15 μg/g in soymilk from 10 cultivars and 23.79–91.03 μg/g in soymilk kefir. Furthermore, daidzein exhibited the highest average concentration, followed by genistein, while glycitein was not detected after fermentation. In particular, soymilk kefir from Guixia 2 (Guangzhou) and Wayao showed higher isoflavone content than soymilk kefir produced from other cultivars, with a threefold increase in aglycone isoflavone daidzein and genistein during fermentation. In contrast, the lowest levels were found in Huaxia 10 variety (23.79 μg/g) and in Huaxia 3 variety (29.21 μg/g) (Figure 3). Interestingly, the soymilk Guixia 2 cultivar from two locations, Yingde and Guagzhou, showed a significant difference in isoflavone content in soymilk kefir. Fermentation processes seemed to increase the concentration of free isoflavone, which might be attributed to the action of microbiota from kefir. Therefore, Guixia 2 (Guangzhou) and Wayao might be good resources for soymilk kefir fermentation because of a higher aglycone isoflavone content. The first one was selected for the next step of our research.

**Table 3:** Changes in isoflavone contents in guixia 2 (guangzhou) soymilk kefir fermented with six different kefir starters.

**Isoflavone**	**Contents of isoflavones (μg/g) in soymilk kefir fermented with six kinds of kefir starters**						
	**CK**	**S1-C**	**B**	**S1-B**	**S2-B**	**S1-A**	**S2-A**
Total daidzein conjugates	117.18 ± 2.11^a^	59.76 ± 2.94^e^	101.48 ± 5.58^b^	93.61 ± 3.44^c^	98.66 ± 4.08^b^	84.28 ± 2.38^d^	80.36 ± 4.61^e^
Total genistein conjugates	27.61 ± 1.24^a^	23.25 ± 0.52^bc^	23.83 ± 0.95^b^	23.79 ± 0.76^b^	22.70 ± 0.93^bc^	21.95 ± 1.26^c^	21.22 ± 0.89^c^
Total glycitein conjugates	362.43 ± 18.07^a^	326.64 ± 6.81^b^	349.94 ± 15.31^ab^	362.17 ± 15.79^a^	348.48 ± 5.65^ab^	340.53 ± 13.96^ab^	333.30 ± 8.24^b^
Total conjugated isoflavone	507.22 ± 20.63^ab^	409.64 ± 9.85^c^	475.25 ± 21.79^b^	479.57 ± 19.89^ab^	469.83 ± 10.54^ab^	446.76 ± 17.51^b^	434.88 ± 13.62^b^
Total individual isoflavone	45.87 ± 3.52^e^	68.30 ± 1.96^b^	54.32 ± 3.14^d^	60.32 ± 1.70^c^	61.55 ± 1.52^c^	63.45 ± 0.57^c^	72.07 ± 0.53^a^

Mean ± standard deviation; n = 3. Means in the same row with different letters are significantly different (*p < 0.05). CK: Soymilk. B: Soymilk fermented with kefir B. S1-A: Soymilk fermented with the addition of 5 mL fermented milk to kefir A. S2-A: Soymilk fermented with the addition of 1.5 g probiotics to kefir A. S1-B: Soymilk fermented with the addition of 5 mL fermented milk to kefir B. S2-B: Soymilk fermented with the addition of 1.5 g probiotics to kefir B. S1-C: Soymilk fermented with the addition of 5 mL fermented milk to kefir C. Different superscript letters (a, b, c, d, and e) indicate significant differences in isoflavone concentration among different soybean cultivars (*p < 0.05).

### Changes in Aglycone Isoflavone Contents in Soymilk Kefir With Five Different Starters

**Figure 4:**
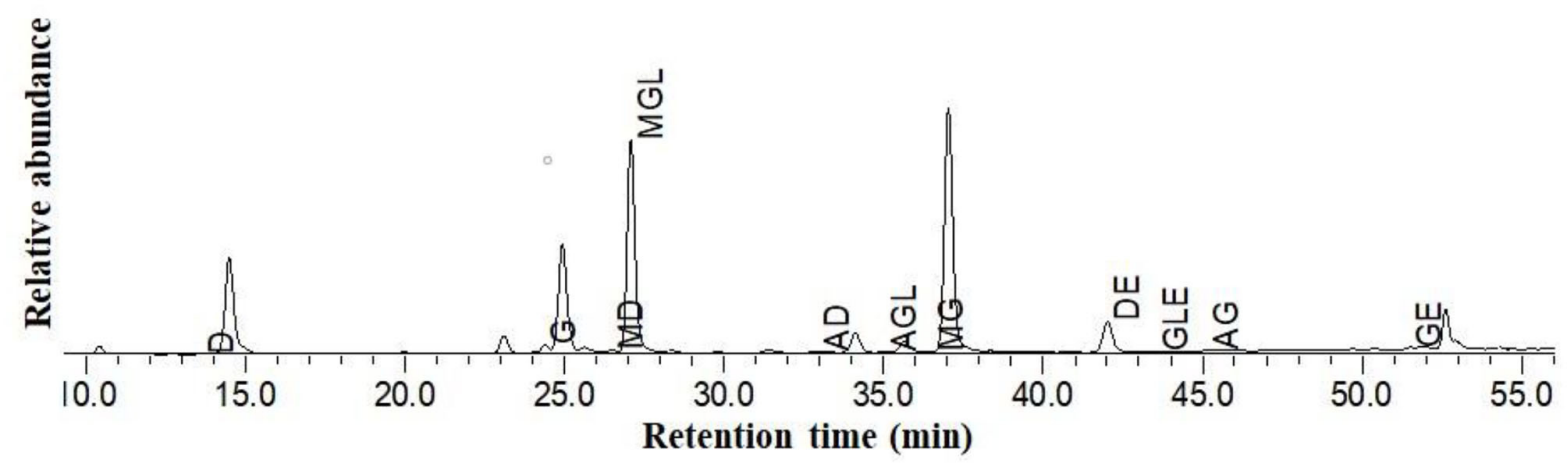
HPLC chromatogram of isoflavones from Guixia 2 (Guangzhou) soy milk. D, Daidzin; GL, Glycitin; G, Genistin; MD, Malonyldaidzin; MGL, Malonylglycitin; AD, Acetyldaidzin; AGL, Acetylglycitin; MG, Malonylgenistin; DE, Daidzein; GLE, Glycitein; AG, Acetylgenistin; GE, Genistein.

Representative HPLC chromatograms of Guixia 2 soymilk (sample CK, from Guixia 2) are shown in Figure 4. Malonylglycitin was found to be the most predominant isoflavone. The remaining types showed different relative abundance in the following order: malonyldaidzin > glycitin > daidzin > genistein > acetylglycitin > genistin > malonylgenistin > daid-zein > glycitein > acetyldaidzin > acetylgenistin.

**Figure 5:**
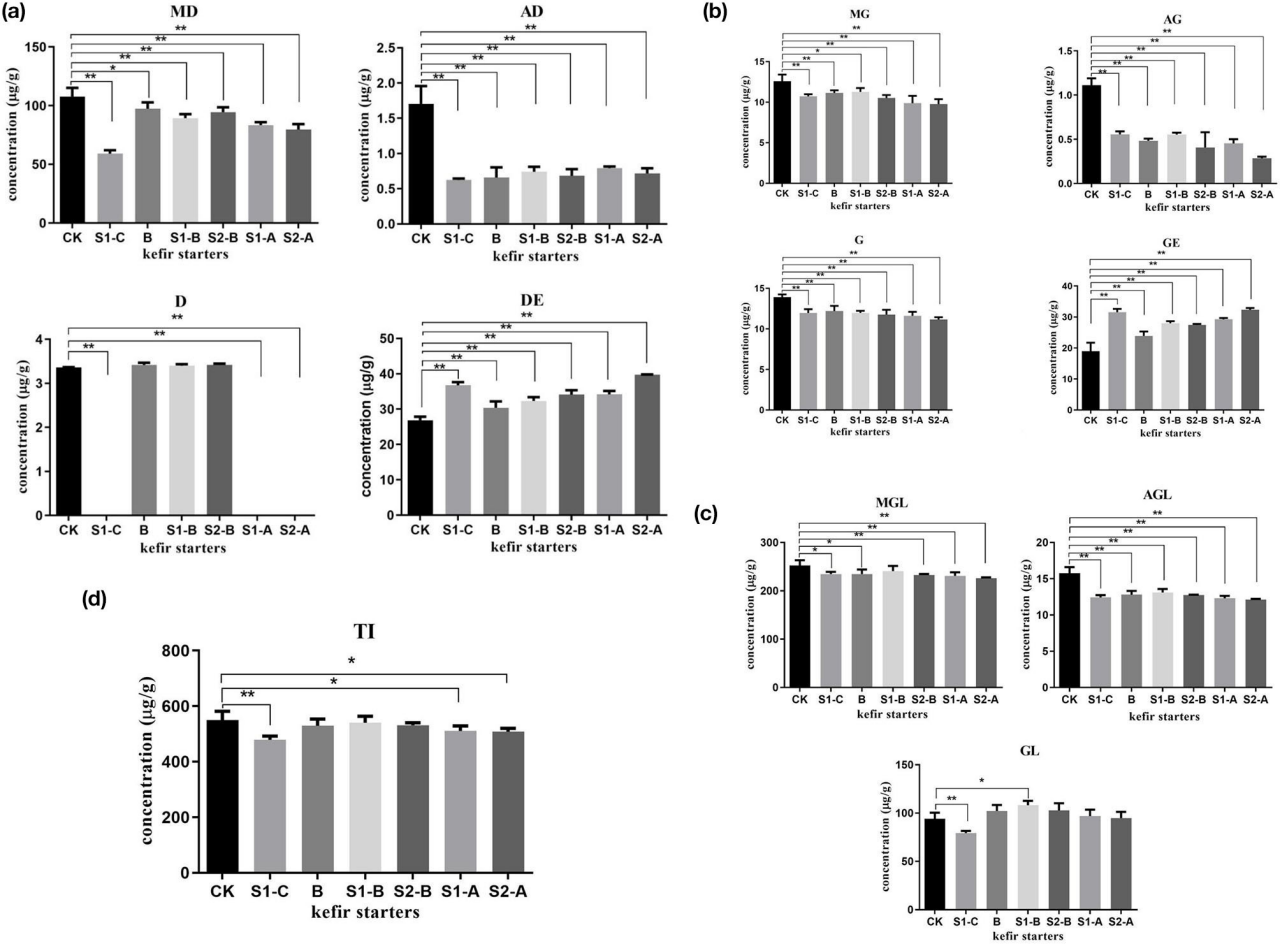
The amounts of glycosides, genistein, glycitein and its conjugates **(a–c)**, and the total isoflavone **(d)** in Guixia 2 soymilk fermented with six different kefir starters. Data are the mean ± SD of three separate experiments performed in triplicate. One asterisk indicates a significant difference (*p < 0.05), and two asterisks indicate an extremely significant difference (**p < 0.01), with respect to Guixia 2 soymilk (the control group). TI, total isoflavone. **(A)** Soymilk fermented with kefir A. **(B)** Soymilk fermented with kefir B. **(C)** Soymilk fermented with kefir C. S1-A: Soymilk fermented with the addition of 5 mL fermented milk to kefir A. S2-A: Soymilk fermented with the addition of 1.5 g probiotics to kefir A. S1-B: Soymilk fermented with addition of 5 mL fermented milk to kefir B. S2-B: Soymilk fermented with addition of 1.5 g probiotics to kefir B. S1-C: Soymilk fermented with addition of 5 mL fermented milk to kefir C.

The isoflavone content of the conjugated daidzein and its free form are illustrated in Figure 5a. Notably, the abundance of malonyldaidzin and acetylglycitin in soymilk kefir decreased dramatically by fermentation, and the presence of daidzin in samples of S1-A, S1-C, and S2-A was not detected.

The concentration of the conjugated genistein was low in soymilk kefir. However, a significant increase in genistein was observed in the S1-C and S2-A samples (Figure 5b).

There was a significant decrease in malonylglycitin and acetylglycitin (Figure 5c) in all fermented samples compared to the control. However, the fermentation of soymilk showed no significant effect on the change in glycitin content except for the S1-C sample, which had a considerable reduction in the amount of glycitin. However, glycitein was not detected in soymilk kefir.

As shown in Figure 5d and Table 3, soymilk kefir showed a high concentration of isoflavone aglycone, whereas the total isoflavone had a small reduction under fermentation.

## Discussion

Our research indicated that Firmicutes, Proteobacteria, and Bacteroidates predominated in soymilk kefir, which was in agreement with some previous studies that confirmed the presence of the three major bacterial phyla. Firmicutes was the dominant phylum, making up 92% or more of the total sequences within the kefir milk ferment (36). Compared to the milk kefir, an increase in bacterial phyla diversity was shown in soymilk fermented with kefir. We can deduce that abundant nutrient ingredients in soymilk, such as fiber and protein, may facilitate the growth of certain microbiota in kefir. As in previous studies, soymilk kefir presented the highest *L. lactis* count value, perhaps because of its different types of protein and soy fiber in soy milk (37).

Good flavor is the most important and basic attribute of a food product. It determines the likelihood of product success in the market. In our study, the score increased from A starters to their modification samples S1-A and S2-A, suggesting that the addition of LAB in Kefir A may improve the aroma and taste of soymilk kefir. As stated above, the difference in flavor contribution during soymilk fermentation was observed in nine samples, which was influenced by their microbial composition before or after LAB modification. Thus, it appears that not all kefir samples were suitable for soymilk fermentation for flavor characteristics.

The aglycone isoflavone content of soymilk kefir varied among different soybean cultivars (Figure 3). In particular, the difference in aglycone isoflavone concentration in soymilk kefir was related to its original concentration in the soymilk from the 10 soybean cultivars. This is consistent with other studies, which showed that the content of aglycone isoflavone in other soymilk products, such as tofu, was also correlated with the soybean cultivar itself (38, 39). The proportions of the total concentrations of aglycone isoflavones varied among cultivars, especially in the malonyl-glucoside group (20), and strong correlations were observed between aglycone isoflavone content in soymilk and soybean cultivars (15). Recently, molecular genetic approaches based on QTL mapping have been conducted to promote the understanding of genome-based isoflavone content (40, 41).

During fermentation, the isoflavone (daidzin) concentrations from different samples varied, depending on the species of starter. Before fermentation, conjugated isoflavones were present in high quantities in the soymilk. After 24 h fermentation, the daidzein and genistein contents were increased more by microorganisms in Kefir A modified with probiotics than others. It was observed that, in this fermentation, β-glucosides and malonyl- and acetyl-conjugated glucoside content were reduced, likely due to the catalysis of β-glucosidase produced by this culture and degraded into aglycones. Daidzin decreased rapidly in soymilk fermented with modified Kefirs A and C but was limited in Kefir B and its modified culture fermentation. This may be due to the conversion of daidzin to daidzein by β-glucosidase produced by the microorganism from the original Kefirs A and C. In addition, the microorganism that originally exists in kefir grains, which is preferentially metabolized daidzin but in very low concentrations (accounting for <1%), proliferates rapidly and shows dominance after the LAB addition. Furthermore, glycitein was not detected in soymilk kefir, indicating that it might be vulnerable to the degradation of microorganisms in large number or it has been converted to isoflavone derivatives other than glycitein. This is consistent with the findings of Lim (42) that glycitein was the most degraded of the aglycones. Moreover, the conjugated isoflavone might convert not only to corresponding aglycones but also to other substances during fermentation, which was evidenced by the decreased total isoflavone contents. These phenomena may be explained as follows. Daidzein can be converted to equol by enzymes of bacteria (43), which is difficult to detect and quantify (44).

Our results also demonstrated that the kefir ecosystem remained quite stable with the addition of exogenous LAB, and the proliferation of these microorganisms may contribute to the particular sensory characteristics of the soymilk kefir (fizziness, acidic taste, and refreshing flavor) via the production of metabolites such as organic acids, ethanol, and aromatic compounds.

In this study, the significant bioconversion of the glucoside isoflavones into their corresponding aglycones during soymilk kefir fermentation was due to cleavage of the glycosyl bond by microbial fermentation. The discrepancy of β-glucosidase production was attributed to the differences in bacterial strains. *Lactobacillus* and *Lactococcus* from kefir may play an important role in the soymilk kefir fermentation. The greatest increase in isoflavone aglycone concentration was observed in soymilk fermented with *Lb. acidophilus* 4461, which detected the highest β-glucosidase activity at 24 h (45, 46). Choi et al. (47) reported that *Lb. delbrueckii* subsp. *delbrueckii* KCTC 1047 hydrolyzed genistin and daidzin completely in soymilk. The content of aglycone isoflavones also increased impressively in the soymilk fermented with *S. thermophiles* (48, 49). Meanwhile, isoflavone glucoside-hydrolyzing enzyme depended not only on the bacterial strain but also on the culture medium, with some lactic acid bacteria produced only in soymilk medium (47). There was no probiotic organism efficient at performing the biotransformation of all three biologically active aglycone isomers. Specifically, glycitein was the highest in soymilk inoculated with *L. paracasei*, and genistein was the highest in soymilk fermented by *L. plantarum* (50). It corresponded to our premise that the combination or facilitation of more bacteria in kefir enhanced the function of soymilk kefir.

This work is the first to consider the selection of suitable soybean cultivar and kefir starter simultaneously during the soymilk kefir fermentation. In the current study, (1) the suitable soybean cultivar was determined based on the higher levels of aglycone isoflavones in its soymilk kefir, and (2) a whole new microbial composition in kefir culture was built by the addition of exogenous LAB. The rebuilt kefir culture showed stronger ability to hydrolyze β-, malonyl-, and acetyl-conjugated glucoside than the original one, which can be reflected in the higher aglycone isoflavone concentration in soymilk kefir. Therefore, it was suitable for soymilk kefir fermentation.

In the near future it can be proposed that the suitable starter culture and soybean cultivar of soymilk kefir can be defined due to their enhancement of aglycone isoflavone contents and pleasant flavor during fermentation. Such a soymilk kefir starter can be used as a functional fermented drink, as well as an auxiliary food for menopausal women during a specific pathological period.

## Data Availability Statement

The datasets generated for this study can be found in European Nucleotide Archive, Accession No. PRJEB40909.

## Author Contributions

XF conceived the study conception. MY and XC performed the experiment. MY drafted the manuscript and the analysis and interpretation of data. JW, ZL, LW, and QZ revised the initial manuscript critically. All authors contributed to manuscript revision and read and approved the submitted version.

## Conflict of Interest

The authors declare that the research was conducted in the absence of any commercial or financial relationships that could be construed as a potential conflict of interest.
